# Simple, Fast and Accurate Implementation of the Diffusion Approximation Algorithm for Stochastic Ion Channels with Multiple States

**DOI:** 10.1371/journal.pone.0036670

**Published:** 2012-05-22

**Authors:** Patricio Orio, Daniel Soudry

**Affiliations:** 1 Centro Interdisciplinario de Neurociencia de Valparaíso, Facultad de Ciencias, Universidad de Valparaíso, Valparaíso, Chile; 2 Department of Electrical Engineering, Technion, Haifa, Israel; 3 Laboratory for Network Biology Research, Technion, Haifa, Israel; University of Antwerp, Belgium

## Abstract

**Background:**

The phenomena that emerge from the interaction of the stochastic opening and closing of ion channels (channel noise) with the non-linear neural dynamics are essential to our understanding of the operation of the nervous system. The effects that channel noise can have on neural dynamics are generally studied using numerical simulations of stochastic models. Algorithms based on discrete Markov Chains (MC) seem to be the most reliable and trustworthy, but even optimized algorithms come with a non-negligible computational cost. Diffusion Approximation (DA) methods use Stochastic Differential Equations (SDE) to approximate the behavior of a number of MCs, considerably speeding up simulation times. However, model comparisons have suggested that DA methods did not lead to the same results as in MC modeling in terms of channel noise statistics and effects on excitability. Recently, it was shown that the difference arose because MCs were modeled with coupled gating particles, while the DA was modeled using uncoupled gating particles. Implementations of DA with coupled particles, in the context of a specific kinetic scheme, yielded similar results to MC. However, it remained unclear how to generalize these implementations to different kinetic schemes, or whether they were faster than MC algorithms. Additionally, a steady state approximation was used for the stochastic terms, which, as we show here, can introduce significant inaccuracies.

**Main Contributions:**

We derived the SDE explicitly for any given ion channel kinetic scheme. The resulting generic equations were surprisingly simple and interpretable – allowing an easy, transparent and efficient DA implementation, avoiding unnecessary approximations. The algorithm was tested in a voltage clamp simulation and in two different current clamp simulations, yielding the same results as MC modeling. Also, the simulation efficiency of this DA method demonstrated considerable superiority over MC methods, except when short time steps or low channel numbers were used.

## Introduction

Noise and variability are present throughout the nervous system, from sensory systems to the motor output and perhaps more importantly in the higher brain areas [1]. Far from being considered as a nuisance, noise is now argued to be one of the key elements that shape the way the central nervous system (CNS) codes sensory inputs, builds internal representations and makes decisions [2]. Phenomena like stochastic resonance [3], [4], [5], [6] enhance several aspects of sensory coding and signal detection [7], [8]. Also, noise can be beneficial in various computational tasks [9], [10], [11], [12].

One of the main sources of noise and variability is the stochastic opening and closing of ion channels, commonly called *channel noise* [13], [14]. The effects of channel noise on neuronal excitability are to a large extent studied with the use of mathematical models, either by constructing and analyzing models with stochastic channels [15], [16], [17], [18] or by introducing a noisy conductances in dynamic clamp experiments [19], [20]. It is of interest, then, to develop and analyze numerical models that faithfully reproduce the stochastic nature of ion channels. It is also of interest to develop fast algorithms that can be used in large scale simulations of neural networks or in real time simulation for dynamic clamp experiments.

Ion channels are commonly modeled using the framework established by Hodgkin and Huxley [21], see also [22]. In this framework, ion channels contain one or more *gating particles* that can be either in a resting or active state. The transition rates between states are voltage-dependent, and now we know that this is because these particles contain a charged domain (the *voltage sensor*) that senses the membrane electrical potential [23]. In the pure Hodgkin and Huxley (HH) framework, the probability of a channel being open is equal to the probability of all its gating particles being active. Usually the particles are assumed to be independent and thus the probability of the open channel is the product of the probabilities of the active particles. In the limit of infinitely many channels (deterministic HH model), probabilities are equivalent to the *fraction* of active particles or open channels. The transition between resting and active states of particles is described by ordinary differential equations of a deterministic nature, because the HH model fitted the behavior of a giant squid axon with such a large number of channels that individual stochastic contributions were completely neglected.

When the stochastic behavior of ion channels is taken into account, it is best described by continuous-time, discrete state Markov jumping processes [24], [25]. Several algorithms exist for the mathematical simulation of simultaneous and independent Markov Chains (MCs) representing a population of ion channels in a membrane patch or neuronal soma. Among these, the most efficient is a channel-number-tracking algorithm proposed by Gillespie [26] and first applied to ion channels in 1979 [27] (see [28] for a comparison with other MC algorithms). Nevertheless, all MC algorithms increase their computational complexity with the number of channels.

Another approach for simulating stochastic ion channels relies on the fact that a large number of simultaneous and independent MCs can be approximated by a stochastic differential equation that describes the time evolution of the fraction of MCs that are in each possible state [29], [30], [31], [32], [33]. This algorithm, referred as Diffusion Approximation (DA), is dramatically more efficient in terms of computational cost [28] and is the choice for dynamic clamp experiments where real-time simulation is required [19]. In the general form of DA [29], the time evolution of a variable vector containing the fraction of channels in each state is obtained by solving a Langevin equation (see eq. (1)) with both deterministic and stochastic transition matrices. The method, however, is less practical, since it requires the numerical calculation of a matrix square root at each time step, making it a very time-consuming algorithm (each calculation usually requires about 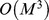 floating point operations [34], 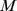 being the number of channel states). To circumvent this, Fox and Lu [29] heuristically proposed to simulate the two-state gating particles as separate stochastic processes and then calculate the conductance of each ion channel species as the product of particle probabilities. This approach of uncoupled gating particles requires a simple Stochastic Differential Equation (SDE) per particle species without any matrix operation, easily constructed by adding simple noise terms to the deterministic differential equations of the mean channel kinetics. This, in addition to its high computational efficiency, made the uncoupled particles approach the main choice for DA implementations [18], [19].

However, the uncoupled particles form of the DA does not approach the behavior of explicit MC appropriately. Mino and colleagues [28] found that this DA algorithm introduces less variability than MC modeling, evidenced by a steeper action potential firing probability vs. stimulus intensity relationship. Later, Bruce [35] found that the DA algorithm, as it was being implemented, assumes that the stochastic term of the gating particles is uncorrelated, while the MC modeling introduces correlated noise into the channel conductance behavior. Also, the variance of the conductance is higher for MCs than for the uncoupled particles DA algorithm.

Why was it assumed that gating particle coupling is of minor importance when modeling stochastic channels? Mainly, because both approaches – coupled or uncoupled particles – result in a similar *mean* time evolution of the conductance [33]. However, fluctuations introduced by both approaches are dramatically different, in terms of the *variance* of the conductances and their *correlations* at different times. This difference between approaches poses a serious problem since the purpose of any quantitative stochastic model is precisely to determine the effects of these fluctuations. The uncoupled particles approach also has the disadvantage of not being applicable to kinetic schemes with non-independent gating particles – such as channels with cooperative voltage sensors [36], [37]– or when the voltage sensors are not identical [38], [39].

In recent works [33], [40], it was further confirmed that considering coupled gating particles produces more variability in the conductance and introduces noise with a particular covariance that cannot be reproduced by two-states models. Both works also proposed algorithms for the DA that better approached the results of MC modeling, in the context of the HH model. Goldwyn *et al.* [33] tested the general form of DA suggested by Fox [29], numerically computing the square root of the stochastic diffusion matrix (an 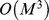 operation) at each time step, producing a very time-consuming algorithm. On the other hand, Linaro *et al.* [40] developed a set of SDEs that capture the statistical properties of the variations of conductance, adding it to the ion currents given by a deterministic model.

Here we present a different approach to derive the DA using basic probabilistic tools, for any given kinetic diagram of a channel. This derivations results in practical, general and intuitive rules allowing for the accurate implementation of DA as a set of simple SDEs, with comparable simplicity to that of (inaccurate) uncoupled DA approach, allowing and efficient implementation (between 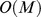 and 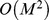 at each time step, depending on the number of kinetic transitions). This makes the computational complexity of the stochastic algorithm comparable to that of the uncoupled DA approach and even the deterministic implementation that simply ignores the noise terms in the SDE. We thoroughly tested the proposed DA implementation, comparing its results to the behavior of explicit MC modeling in three different simulation tests: one under voltage clamp and two under current clamp. Notably, the methods previously suggested [33], [40] displayed significant inaccuracies in two of these tests because they employ a steady-state approximation for the calculation of stochastic coefficients. Our method does not require such an approximation and therefore does not incur those errors. We also compare the computational efficiency and numerical stability of the algorithm for different numbers of channels and integration time steps, showing that in most cases DA will be algorithm of choice. Finally, we discuss how our method relates to other implementations previously published.

## Results

### Mathematical Analysis

We examine a specific population of 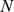 ion channels with 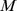 states, where the transition rate of a single channel from state 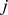 to state 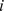 is given by 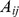. We define the rate matrix 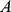 to be composed of these 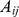 terms for all 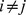, and also 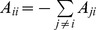 on the diagonal. In neuronal models, these transition rates are usually voltage dependent (and so are also time-dependent). For brevity, we keep this voltage dependency implicit. We denote by 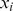 the fraction of channels in each of the state, and by 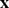 a vector of 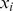. Note that 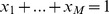 and it is common to use this normalization in order to reduce the number of variables [29], [31], [33], [40]. However, here this substitution is not employed until the numerical implementation to make the algebraic operations easier. The DA proposed by Fox [29], [31] for the stochastic dynamics of 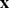 leads to the following SDE.
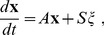
(1)where 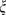 is a vector of independent Gaussian white noise processes with zero mean and unit variance, 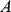 is the rate matrix, and 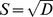, a square root of the diffusion matrix 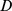 (namely 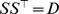). This matrix square root has been the main hindrance in the implementation of DA [33]. If solved numerically in simulation time, it incurs a great computational cost, of order 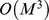 at each time step.

Interestingly, it is possible to obtain a direct analytical solution of 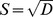 for certain kinetic schemes, such as the potassium channel scheme, prior to the simulation (we used Cholesky decomposition, see eq. (15) and below). However, it is not immediately clear how to do so for other schemes, such as the sodium channel scheme. We therefore explored a different derivation of the matrix 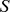.

#### Derivation of the diffusion approximation

We denote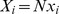, the number of channels in state 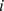, and 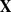 to be the corresponding vector. Assume that 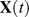 is known, and we wish to calculate 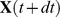. Recall that the channels are independent of each other and that transition rates are memoryless. Therefore, for all 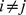, we define the channel transition step.
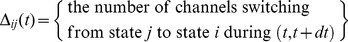
(2)



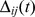
 is a Random Variable (RV) composed of the sum of 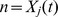 independent events (“trials”), in which a channel either switched states, with probability of 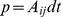, or did not switch states, with probability 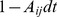 (to first order in 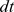). This entails that for all 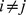, 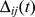 are independent and binomially distributed with 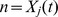 and 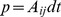. Denoting by 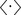 the expectation (over the ensemble), we use the properties of the binomial distribution and find the mean.
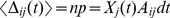
(3)and the variance,



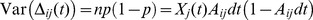
(4)Since 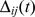 are independent.
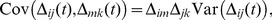
(5)where 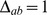 if 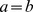, and 0 otherwise.

In the limit 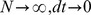 we get that 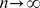 and 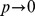 for the binomial distribution of each 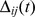. This allows us to approximate 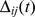 by a normal (Gaussian) distribution with both mean and variance equal to 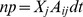 (by the central limit theorem). In order to derive the SDE (eq. (1)), we need to assume that the Gaussian approximation is reasonable. Later, we confirm this numerically, as also did Linaro *et al.* [40] and Goldwyn *et al.* [33] (for example, this was numerically confirmed by [33] for channel numbers as low as 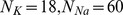).

At each 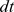, 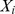 changes according to the sum of channels entering and leaving state 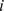.
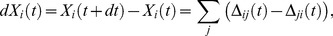
(6)


where we defined, for convenience, 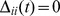. Assuming 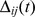 are all normal, then 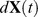 (the vector of 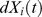) is also normal, as a linear combination of independent normal RVs. Since the distribution of normal variables is entirely determined by their mean and covariance, we calculate them.

We use eq. (3) to find the mean of eq. (6).
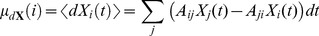
(7)


Next, using eq. (5) we find the covariance.
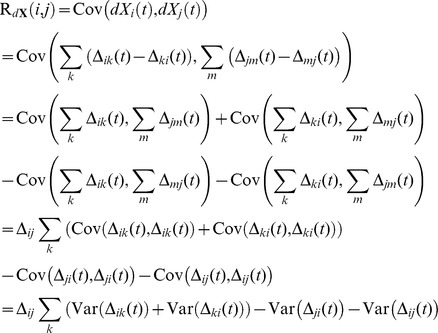



Using eq. (4), neglecting *dt* ^2^ terms and dividing by *dt* we obtain.
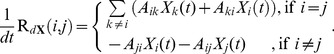
(8)


Since we now know the mean of 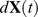 (eq. (7)) and the covariance between all of its components (eq. (8)), we can write.
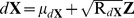
(9)where 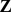 is a vector of independent Gaussian RVs with mean zero and unit variance. To derive an SDE for 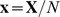
we divide eq. (9) by 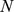 and take the limit of 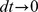, yielding.



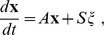
which is indeed eq. (1), with 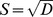, where


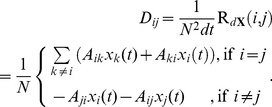
(10)

### A Simpler Derivation of the Diffusion Approximation

Now that we have the general expression for the diffusion matrix, and know its origin, we can devise a simple way to explicitly calculate 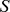, which avoids the use of time consuming numerical procedures for matrix square root computation. The key idea behinds this is to use only 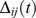 and eqs. (3)-(6) to derive the SDE, and the Gaussian approximation. For simplicity, we demonstrate this method step-by-step using a channel with 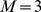 states.
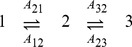



Using eq. (6) we write
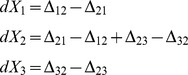
(11)


Denoting 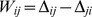 we notice that 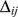 can be combined in opposing pairs.
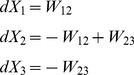
(12)


We now calculate the means, using 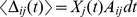 (eq. (3)), we obtain.
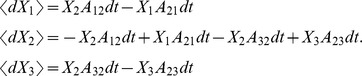



Denoting 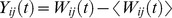, we obtain.
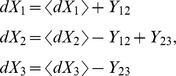
where 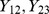 are normal, independent, with zero mean and



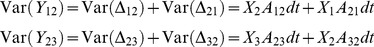
,where we used eq. (4), neglecting 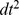 terms. Now we can write



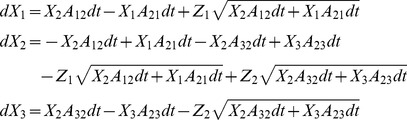
with 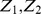 are normal, independent, with zero mean and unit variance.

Dividing by 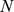 and taking the limit 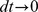, we finally obtain the SDE.
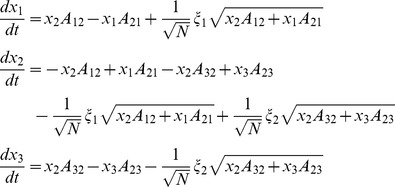



Note that each component of 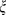 is associated with a transition pair 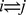, multiplied by 
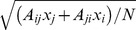
, and appears in the equations of 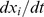 and 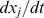 with opposite signs.

Using a similar derivation we can now write 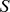 for a general channel with 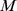 states. To do this succinctly we must introduce several notations. We denote by 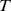 the set of all possible transitions pairs 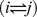 that exist between states and then give each pair an index in 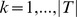. Note that 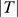, the size of set 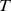, can be any integer between 0 and 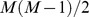. Also, we denote 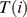 to be the subset of all transitions pairs that connect to state 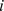. Finally, we denote 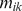 to be the index of the state connected by the 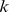-th transition pair, excluding state 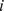.

In that case, the matrix 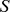 is of size 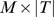, and.
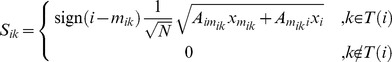
(13)


#### Test case – potassium and sodium channels

We have obtained the matrix 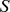 analytically, showing that it has a rather simple structure. It is necessary, however, to compare our result with previous definitions of the diffusion matrix as given by Fox [29], [31] and used by Goldwyn [33]. For a simple comparison, we will use the case of the potassium channel:
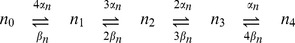



Starting from eq. (1) and defining 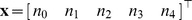, the matrix 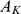 is.
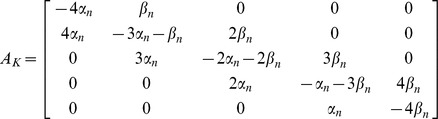




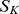
is defined such that 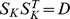 [29], being.
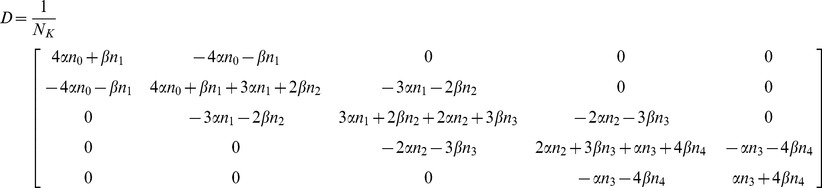
(14)


(*n* sub indices in α and β were omitted for abbreviation). Using Cholesky decomposition, we can find 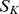:

Substituting in (1) and performing the matrix operations, the full system of SDE for the 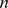


 Substituting in (1) and performing the matrix operations, the full system of SDE for the n. variables can be now written as:
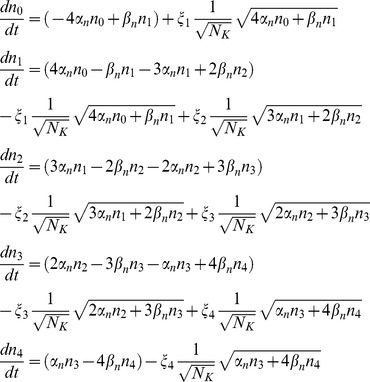
(16)where, again, 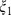, 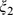, 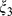
and 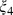 are independent Gaussian white noise terms with zero mean and unit variance. Note in (16) that although the length of the noise vector 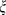 is equal to the number of states, the number of noise terms actually employed is equal to the number of transition pairs 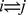. Also, as before, each component of 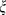 is associated with a transition pair 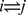; it is multiplied by 
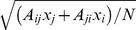
, and then added to deterministic differential equations of 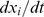 and 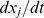 with opposite signs. Thus, the structure of equations we proposed is also obtained from the original definition of 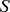.

However, it is easy to see that Cholesky decomposition, which generates lower triangle matrices, will only work for “linear” kinetic schemes – 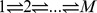. For a example, since a triangle matrix must be square the Cholesky decomposition cannot work if 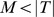, as in the case of the sodium channel, where 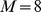 and 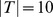. In that case, the 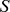 matrix we derive is different than that suggested by Fox [29], [31] and used by Goldwyn *et al.* [33] – since in the latter approach the length of 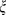 was always equal to 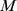, the number of states and not the number of transition pairs, as in our approach. With our approach, the SDE for sodium channels (see Information S1) requires the use of 10 random terms instead of 8 (or 7, if the normalization of 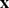 is used). The use of more stochastic terms may appear computationally more expensive, but it comes with the benefit of simple stochastic equations that avoid complex matrix operations. Finally, it is noteworthy that the 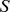 matrix that we propose, with size 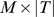, also fulfills 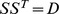, even if 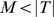.

### Numerical Simulations

In this section we will demonstrate that our equations faithfully reproduce the results that can be obtained in simulations with explicit MCs, with similar numerical stability and lower computational cost. To test the proposed DA algorithm, it was compared to MC modeling in its coupled particles approach. Additionally, we examined a common “steady state” approximation employed when using DA methods. In this approximation the variable values in the expressions multiplying the noise terms are replaced by their steady state values [29], [31], [33], [40]. Here we will show that the steady state approximation must be used with great caution depending on the kinetics of the channels simulated.

The details of the specific models we used and the numerical implementation are described in *Methods*. Before we give the simulations results, we clarify a few important numerical issues.

#### Numerical implementation issues

An issue that is commonly debated in the implementation of DA is whether to manipulate the state variables to make them increase discretely or to bound them between 0 and 1. Mino et al. [28] did both, making the variables to represent an integer number of open channels by multiplying by the number of channels and then rounding them to the lowest integer. Later, Bruce [41] found that rounding to the lowest integer produced a shift of the Firing Efficiency curves to the left, and that it was more appropriate to make the rounding to the nearest integer. In both works the state variables were bounded between 0 and 1 (or between 0 and the number of channels), something that does not impose any mathematical difficulty when dealing with two-state gating particles.

However, when working with multi-state channels, bounding the variables by manually correcting an off-bound value causes the variable vectors to leave a bounded hyperplane that may cause the diffusion matrix to be no longer positive semi-definite, making it impossible to calculate its square root [33]. Therefore, Goldwyn and colleagues decided not to bind the variables and allowed values below 0 and above 1 and instead replaced the variable values in the random terms with their steady state values. We will show here that in some important cases this steady state approximation can introduce significant deviations compared to the exact equations.

In the present work, neither the variables were converted to an integer number of channels nor were they bounded between 0 and 1. The only manipulation performed to ensure real valued random terms was to apply the square root to the absolute value of the argument. As evidenced by the simulations presented here, this did not introduce any noticeable deviation from the simulations with MCs.

#### Voltage clamp simulations

**Figure 1:**
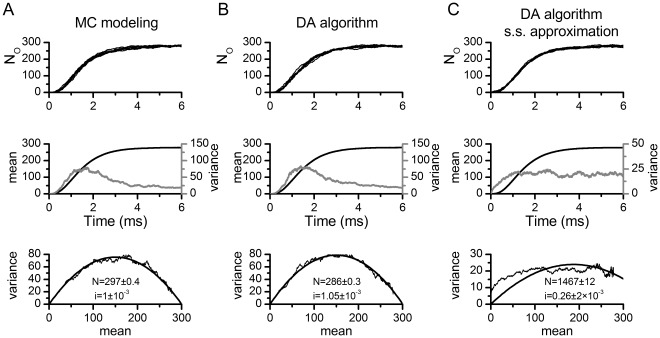
Voltage clamp simulation and non-stationary noise analysis. 300 potassium channels from the HH model were simulated at a constant voltage of 70 mV. At t = 0, they were in a steady state condition calculated at –90 mV. 200 independent simulations were performed with each simulation algorithm (indicated above each panel) and a non-stationary noise analysis was performed [42]. Top row: 8 sample traces of the number of open channels against time. Middle: Mean (black) and variance (grey) of number of open channels as a function of time. Bottom: The variance of the number of open channels is plotted against the mean. The continuous line represents the best fit to equation (17), and the best fit parameters are indicated ± standard error. Expected values are N = 300 and i = 1. The corresponding R-square values are A: 0.98, B: 0.99, C: 0.13.

The behavior of the simulation algorithms was first compared in voltage clamp simulations, using only the potassium channel from the HH model. The initial condition was the steady state value at –90 mV and a 6 second simulation of 300 K channels was performed with the kinetic constants fixed at +70 mV. The number of open channels was recorded at every time step of the simulation (Figure 1A, top, shows 8 simulated traces). 200 independent pulses were simulated and the mean and variance of open channels was calculated for every time step. Figure 1A, middle, shows mean and variance as a function of time and Figure 1A, bottom, shows the relationship between mean and variance of the number of open channels. The relation of the mean and variance of the total current is [42]:
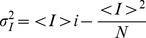
(17)where 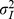 is the variance of the current at any given time, 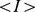 is the mean of the current at the same time, *i* is the single channel current (equal to 1 when counting number of open channels) and *N* the number of channels. This relationship stems directly from the fact that the current in voltage clamp is the sum of independent binary channel currents. In this case, if 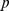 is the probability of finding a channel open, then 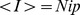 and 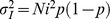, which jointly give eq. (17).

Comparison of Figures 1A and 1B shows that our DA perfectly reproduces the behavior of MC simulations. In both simulations the fit of the data to eq. (17) yields the expected values of *N* and *i.*

The steady state approximation requires the kinetic constants to change slowly compared to the variables. As the kinetic constants are voltage-dependent, the voltage has to change slower than the variables. In a voltage clamp simulation, exactly the opposite happens as the voltage is changed instantaneously at time 0. As expected, the algorithm that uses the steady state approximation performed very poorly (Figure 1C). An almost constant variance of the number of open channels was obtained, and the maximum during the rising phase of the mean was lost. As a result, the model did not recover the correct parameters in the mean vs. variance fit.

Thus, our proposed DA algorithm produces the same results as MC modeling and significant differences appear when steady state approximation is used. We will test it further with current clamp models also assessing the numerical stability and processor time cost.

#### Mammalian node of ranvier model

**Figure 2:**
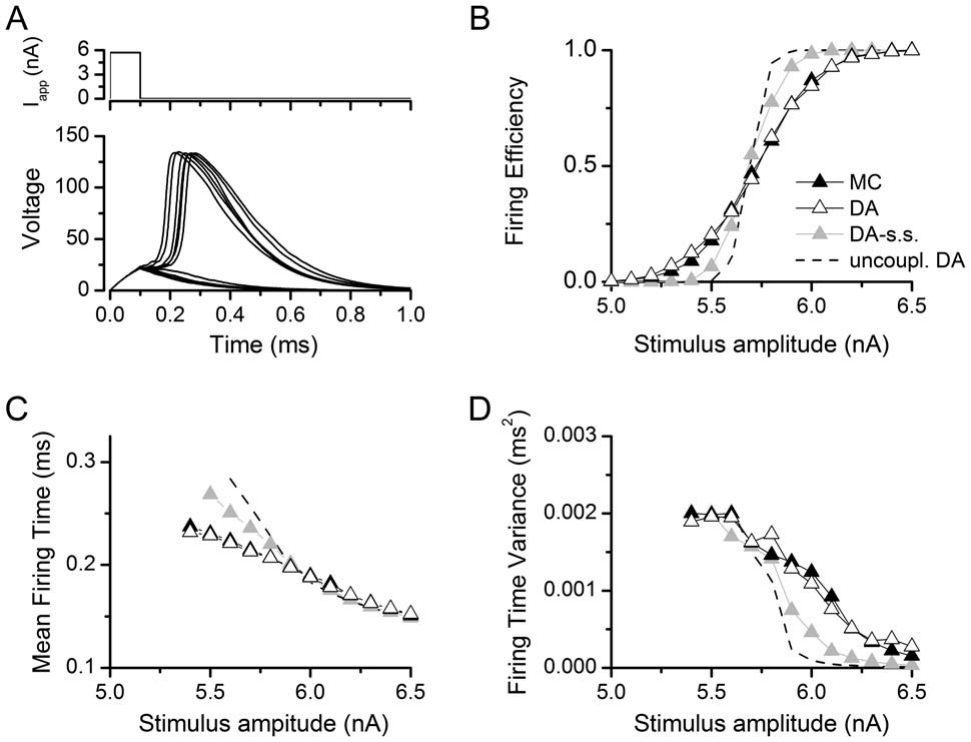
Rb model simulations. A. 15 voltage traces (bottom) resulting from independent simulations with the Rb model, in which a 5.8 nA pulse of 100 µs duration (top) was applied. The simulations presented correspond to the Rb8 model (independent channels approach) using MC modeling, with 1000 Na channels and dt = 1 µs. **B–D.** Firing efficiency (fraction of action potentials evoked in 1000 simulations), mean firing time, and variance of firing time as a function of stimulus amplitude for the different simulation methods. MC: Markov chain modeling, DA: Diffusion approximation algorithm. DA-s.s.: Diffusion approximation with steady state values of variables in random terms. N = 1000, dt = 0.1 µs. For the data plotted in C and D, additional simulations were run for amplitudes between 5.4 and 6.0 nA up to complete 1000 action potentials for a better estimation of mean and variance of firing time. For comparison purposes, dashed lines represent the results of the uncoupled version of the DA algorithm (For method, see Information S2).

The performance of the simulation algorithms in the mammalian Node of Ranvier (Rb) model [43] was tested using a 1 ms simulation in which a single current pulse of 0.1 ms duration and variable amplitude is given at the beginning (Figure 2A). 1000 simulations were performed at each current amplitude level and the measures of action potential variability (defined in Methods, Rb model) are presented in Figures 2B –2D. The curves clearly overlap, indicating the accuracy of our algorithm.

**Figure 3:**
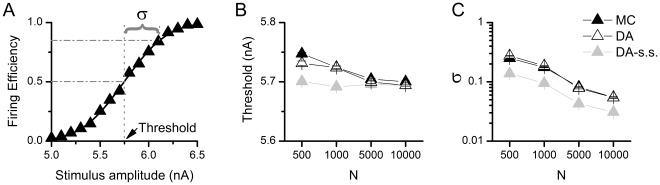
Quantification of variability in the Rb model and its dependence on the number of channels simulated. A. Fitting of a firing efficiency curve to a sigmoid function (see Methods) that is characterized by a threshold (the stimulus amplitude that produces a firing efficiency of 0.5) and σ (the standard deviation of the threshold fluctuations). **B–C.** Dependence of the threshold (B) and slope (C) values on the number of channels simulated, for each of the simulation methods. dt = 1 µs.

While results in Figure 2 correspond to simulations performed with 1000 channels, simulations were also performed with 500, 5000 and 10000 channels. To present the data in a more concise way, the Firing Efficiency vs. Stimulus amplitude curves were fitted to a cumulative Gaussian distribution (Figure 3A). The mean of the distribution corresponds to the *Threshold*, the stimulus amplitude that has a probability 0.5 of firing an action potential, while the standard deviation (σ) is a measure of the spread or the input/output relationship. Figure 3B shows the fitting parameters obtained with different number of channels and the tested algorithms. The most relevant observation in these figures is that DA reproduces the same behavior that is obtained with MC simulation. Also it is interesting to note that the threshold is almost independent of the number of channels, while σ is highly dependent on it. The latter fact is not surprising as fewer channels imply a noisier, more variable simulation and thus a flatter relationship between stimulus amplitude and Firing Efficiency. When more channels are present, noise is reduced and the curve gets steeper, becoming a step function in the deterministic limit (infinite number of channels).

Figures 2 and 3 also show the performance of the DA algorithm with the steady state approximation (grey symbols). With this approximation, the model deviates considerably from the exact algorithm, with less variability as evidenced in the lower spread of the activation curves (σ values). Therefore, it seems that the action potential in the Rb model is fast enough to make the steady state approximation not suitable for a model with coupled gating particles. Finally, we show the inaccurate uncoupled DA in Figure 2, for comparison purposes. The implementation method for the uncoupled version appears in Information S2.

**Figure 4:**
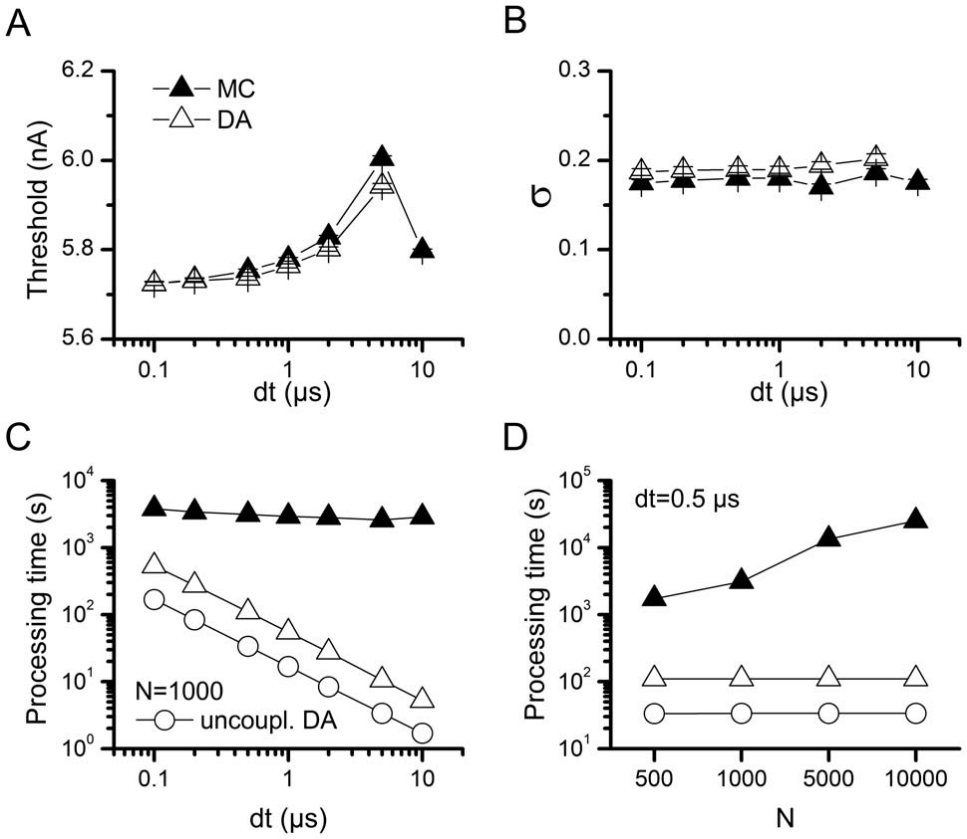
Numerical stability and computational cost of the simulation algorithms with the Rb model. A–B. Dependence of Rb model variability on the integration time step used in the simulation. Threshold (**A**) and σ (**B**) values calculated as in Figure 3A, obtained at different values of integration time step (dt). N = 1000. **C.** Dependence of computation time on integration time step (dt) with N = 1000 channels. **D.** Dependence of computation time on number of channels (N) with dt = 0.5 µs. Computation time is the time, in seconds, needed to perform the 16000 simulations necessary for a single firing efficiency curve (1000 pulses at 16 current levels). This figure corresponds to simulations performed in the Scilab numerical computation software. 1000 simulations were performed as 10 batches of 100 simultaneous and independent simulations, in a Core i7 machine. For comparison, the computation time for the uncoupled version of DA is also depicted.

To test and compare the numerical stability of the algorithms presented here, simulations were performed with increased time steps and the effect of time step on the Firing Efficiency curve was observed. Figure 4A shows that as the time step is increased the threshold also increases, indicating a shift to the right of the Firing Efficiency curve. At dt = 10 µs, there is a sudden drop in threshold, but this is probably a sign of a major instability occurring in the numerical integration. An important observation, however, is that both algorithms show the same behavior, reinforcing the idea that our DA algorithm reproduces the behavior of MC modeling. The spread of the Firing Efficiency curve (Figure 4B) remains to a great extent unchanged as dt is increased and once again the simulation algorithm (MC or DA) does not make any difference. It should be mentioned that when using DA there was a significant number of simulations with dt = 5 µs in which an out-of-range voltage value (NaN, ±Inf) was obtained, and all simulations ended out-of-range for dt≥10 µs. This is to some extent avoided if the variables are constrained to be between 0 and 1, but it comes with some computational cost. Normally, this constraint was not imposed in the simulations presented here (nor in the HH model) and for dt≤1 µs it was not necessary at all. Depending on the kinetics of model to be implemented a decision has to be made as to whether it is worth to add a couple of lines of code that will check and correct values out of boundaries.

Figure 4C–D plots the time it takes to run 16000 simulations (1000 simulations per stimulus amplitude) in the machine employed for this work, as a function of the integration time step (4C) or the number of channels simulated (4D). It is clear that MC modeling is slower than DA, at all conditions tested. We also show the data for the uncoupled version of DA algorithm, to show that our equations approximately just double the computational cost. However, we remind the reader of the inaccuracy incurred by the uncoupled version (Figure 2 and [28], [30], [35], [40]). Another remarkable observation from Figure 4 is that MC modeling is highly affected by the number of channels in the simulation (more channels imply more transitions to calculate) while the DA method is only sensitive to the time step and completely unaffected by the number of channels.

#### Squid axon model

The original Hodgkin and Huxley (HH) model for squid giant axon [21] is deterministic and the channel activation functions are continuous variables. In the absence of a stimulus, no action potential is elicited and the system relaxes to a resting voltage very close to –65 mV. However, if discrete stochastic channels are considered spontaneous action potentials arise due to sodium channels fluctuations [16]. Here, the stochastic HH model was simulated with both algorithms and the resulting spike frequency and intervals were analyzed.

**Figure 5:**
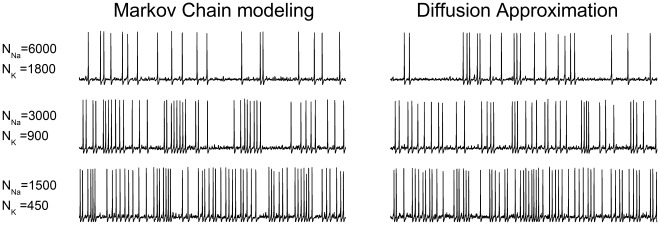
Spontaneous firing in the Hodgkin and Huxley squid axon model. Sample voltage traces of 2 seconds of simulation of the stochastic HH model with the simulation algorithms tested.

**Figure 6:**
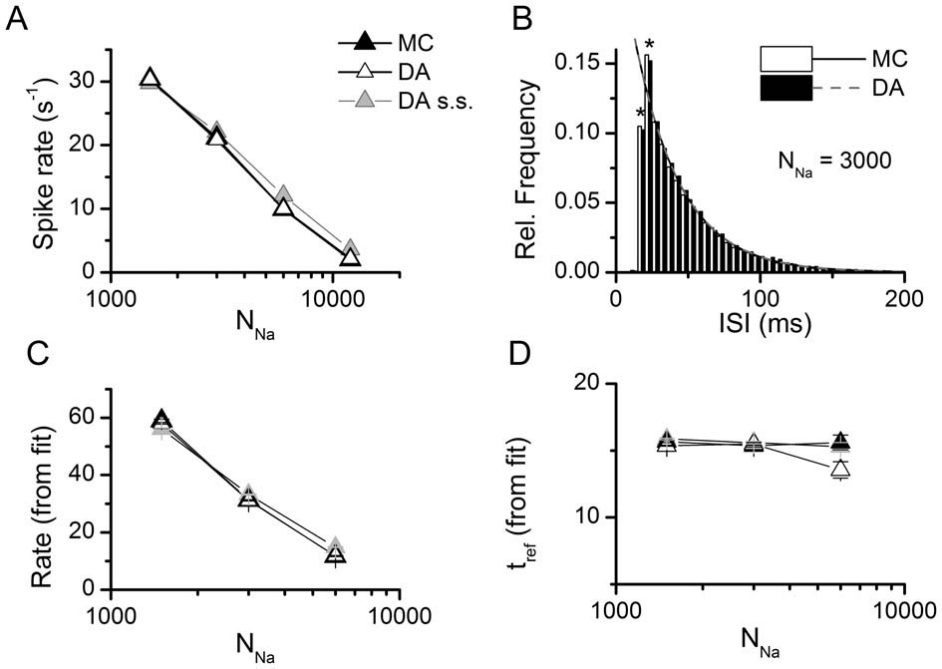
Firing rate and ISI distributions for the stochastic HH models. A. Mean firing rate of the stochastic HH models in a 500 seconds simulation with different number of channels. Note that the symbols for DA and MC superimpose perfectly. **B.** An inter-spike interval (ISI) was built for each simulation and the data was fitted to an exponential decay function with a refractory period (see Methods and ref. [16]). The histograms for only two simulations are shown here for illustration purposes. The first two points (marked with asterisks) were omitted in the fitting procedure (see text). The fit lines for the two histograms showed here overlap almost perfectly. **C–D.** Fit parameters of the ISI distributions at different number of channels. In all the simulations, N_K_ = 0.3×N_Na_. Data in this figure correspond to dt = 0.1 µs.

As expected, the frequency of the spontaneous action potentials increases as the number of channels is decreased (Figure 5). Importantly, our DA algorithm produces the same firing rates as the MC modeling. Figure 6A plots the mean action potential frequency observed in the 500 s simulation, as a function of the number of sodium channels (*N_Na_*) simulated (the number of potassium channels was always set to *N_Na_*×0.3). The result observed with the Rb model is repeated as the simulation algorithm makes no difference in the results. In order to go beyond the simple firing rate quantification, the Inter-Spike Intervals (ISIs) obtained in each case were plotted in histograms and fitted to an exponential decay function (Figure 6B, also see Eq. (22) in *Methods*). For all ISIs obtained, it was observed that the first two bins (marked with * in the histogram) did not follow the exponential trend so they were excluded when fitting the histograms. This was observed in all simulations and thus it is not caused by a specific simulation algorithm. Indeed, it has been observed before [18] and is probably due to the resonant properties of the HH model [21], [44], [45] that, with a frequency of peak response of 67 Hz, will increase the probability of ISIs around 33 ms. Figures 6C and 6D show the fit parameters obtained as a function of the number of sodium channels, and it is evident that the simulation algorithm employed does not make any difference in the ISI distributions.

As with the Rb model, a DA approximation algorithm was tested in which the variable values of the random term were replaced by their steady-state values. The results obtained are plotted in Figure 6 as well (gray triangles). Here the deviations from the exact DA (and MC) are minor, probably because the voltage dynamic in this model is slow enough to let the variables (at least the *m* variable) to be at its steady state value during almost all the simulation.

**Figure 7:**
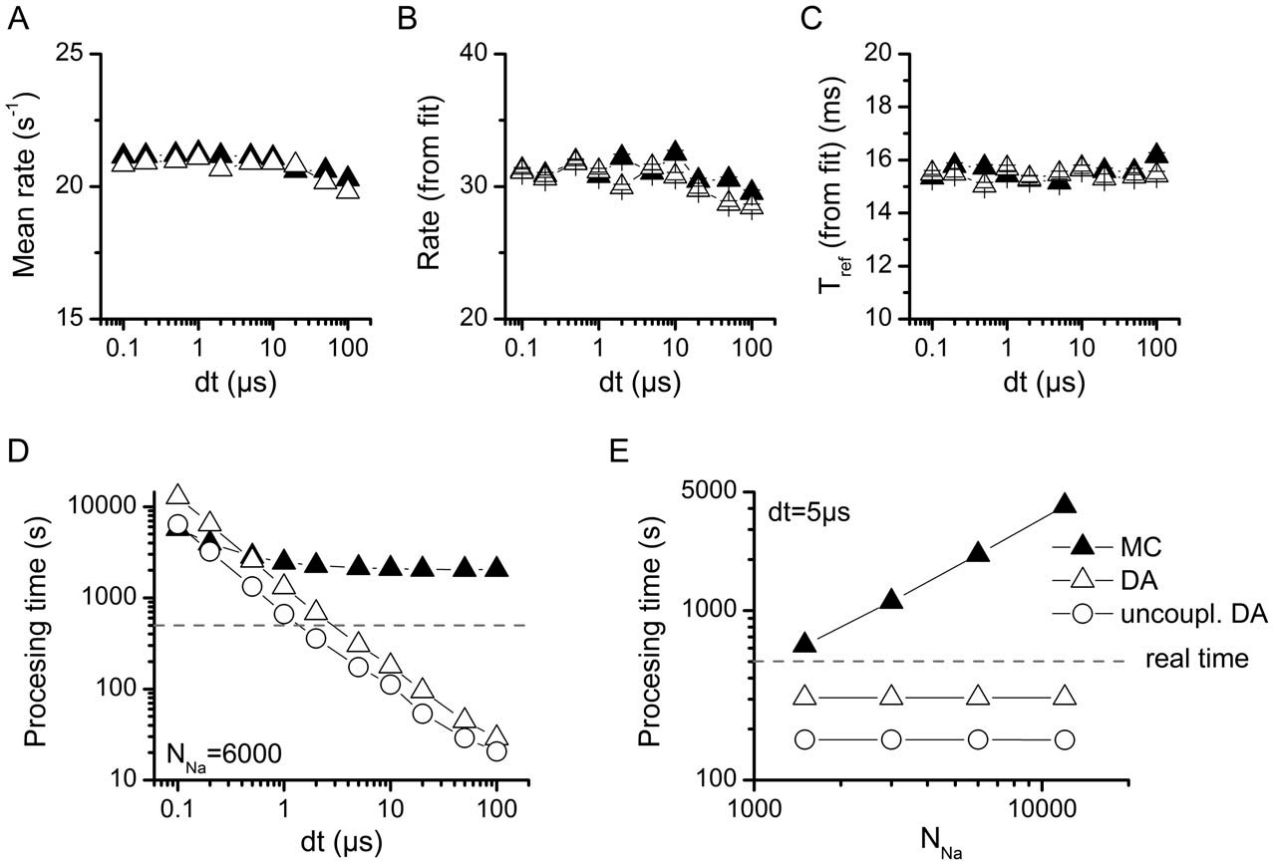
Numerical stability and computational cost of the simulation algorithms with the HH model. A-C. Firing parameters of the stochastic HH models at different integration time steps. Mean firing rate (**A**) and fitting parameters of the ISI distributions (**B–C**) for the stochastic HH models tested as a function of the integration time step (dt). N_Na_ = 3000 and N_K_ = 900. **D.** Time to perform a 500 seconds simulation with N_Na_ = 6000 and N_K_ = 1800 as a function of dt. **E.** Time to perform a 500 seconds simulation with dt = 5 µs as a function of N_Na_, the number of Na channels. N_K_ = 0.3*N_Na_. The segmented line indicates the 500 seconds limit; any simulation below this line runs faster than real time. For comparison, the computation time for the uncoupled version of DA is also depicted.

To check for numerical stability of the methods, simulations were repeated with increasing values of dt, the integration time step. As shown in Figure 7, increasing dt up to 100 µs has little or no effect in the mean rate of spikes (7A) or the parameters of the ISI distribution (7B and 7C). There are some deviations for dt >10 µs, but they are minor compared to what was observed with the Rb model. In this case, no out-of-range voltage values were produced throughout the 500 seconds simulated. Remarkably, the choice of the algorithm has no effect on the numerical stability within the dt values tested.

Figure 7D-E plots the time it took to simulate 500 seconds as a function of the time step (7D) and the number of sodium channels (7E). As with the Rb model, MC modeling performance is severely affected by the number of channels while the DA algorithm is independent of it and only affected by the integration time step. However, in this case MC modeling turned out to be as efficient (in some cases more efficient) than DA at the lowest dt values. This is probably due to the longer time constants of the HH model (reproducing the behavior of squid axons at 6.3°C) compared to the Rb model (mammalian node of Ranvier at 37°C). In the HH model, there are fewer transitions per time step and probably when dt<1 µs there are many steps in which no transition occurs, thus leaving all the computational weight to solving the membrane current equation. However as dt increases more transitions per step begin to occur and then the computational cost is dominated by the calculation of transitions rather than by the advancing of time steps. Again we show the data for the uncoupled version of DA algorithm, to show that our equations approximately just double the computational cost.

#### Accuracy of alternative DA implementations

Two works recently proposed DA implementations that take into account particle coupling [33], [40]. Goldwyn and colleagues [33] tested the DA approach for coupled particle originally developed by Fox [29], and solved the square root of the stochastic diffusion matrix numerically at each time step. Besides the computational cost of this approach, it demands the matrix 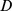
(eq. (10)) to be always positive semi-definite to compute real valued square roots. One simple solution for this, and the one they took, is to use the steady state approximation, replacing the values of the variables by their equilibrium values. On the other hand, Linaro et al. [40] deduced the covariance of the noise introduced by channel fluctuations and showed that it can be reproduced by a sum of Ornstein-Uhlenbeck processes (4 for potassium channels, 7 for sodium channels) with particular time constant and variance coefficients. This noise is then added to the sodium or potassium current, respectively, that are calculated by deterministic Hodgkin-Huxley equations. Importantly, they calculate the noise coefficients using steady-state approximation.

**Figure 8:**
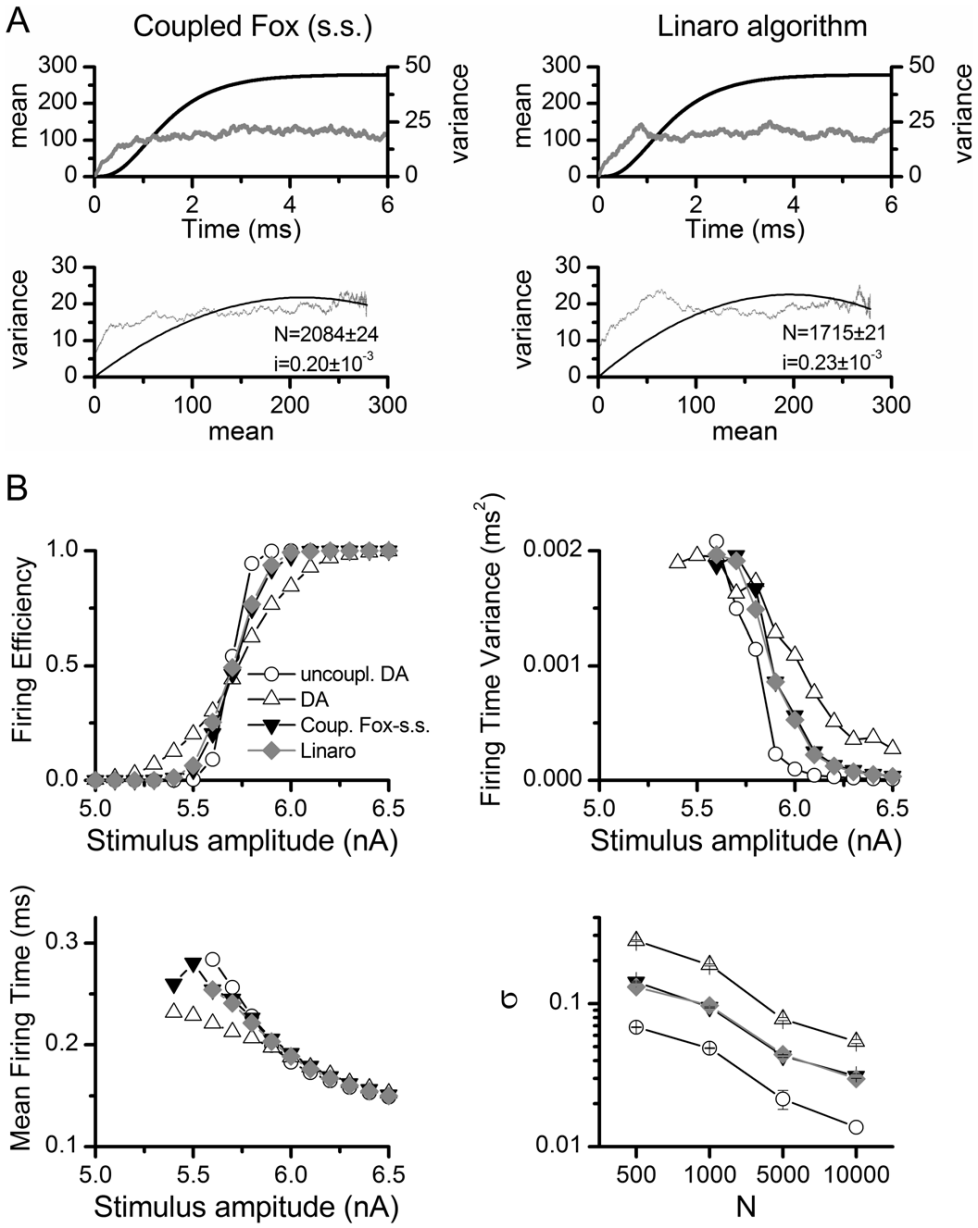
Inaccuracies introduced by previous DA algorithms. A. Performance of the of the Fox [29] algorithm for coupled particles employed by Goldwyn *et al.* [33] and the Linaro *et al.* algorithm [40] in the voltage clamp simulation and non-stationary noise analysis. See legend of Figure 1 for further details. Adjusted R-square values are 0.26 (Fox) and 0.34 (Linaro) **B.** Performance of the algorithms in the Node of Ranvier model simulations. Firing Efficiency, Firing Time Variance and Mean Firing Time versus Stimulus Amplitude are presented for simulations with N_Na_ = 1000. Standard Deviation for Threshold (σ) is plotted against number of channels (see Figure 3). dt = 0.5 µs.

As shown before, the use of a steady-state approximation can result in serious deviations from the explicit MC modeling because the fluctuations become independent on the actual value of the variables at the corresponding time. Figure 8 shows that indeed this is the case, with both algorithms falling short of reproducing the behavior of Markov Chains in the voltage-clamp simulations (note the resemblance of Figure 8A with Figure 1C) as well as in the firing efficiency and firing time variance curves of the Node of Ranvier model (Figure 8B). Also, we show for comparison the inaccurate uncoupled DA version. We managed to implement Fox’s equations without the steady-state approximation, just by extracting the absolute value of the variable vector prior to the matrix square root operation. In that case, the simulations give the same results as MC modeling and our DA implementation (not shown). Therefore, the matrix equations originally proposed by Fox and Lu are indeed a good numerical approximation to MC modeling although with a high computation cost – at least 20 times slower than our method in cases we examined.

## Discussion

### Accuracy of the Diffusion Approximation

The original description of the Diffusion Approximation (DA), in its general form for a multiple (more than 2) state Markov Chain (MC), implies the calculation of the square root of a matrix [29], [31]. As this is too time consuming to be performed in real time, the uncoupled particles approximation, consisting a stochastic form of the original Hodgkin and Huxley’s equations, seemed to be the right choice. Very recently is was described [35] and mathematically proven [32], [33], [40] that when the gating particles are considered to be coupled or ‘tied’ in groups (as they really are in ion channels), the resulting conductance fluctuations have statistics that cannot be adequately reproduced with an uncoupled DA algorithm. As suggested analytically by previous works [33], [40], the uncoupled DA actually approximates a MC with independent and uncoupled gating particles, where the fraction of open channels calculated as the product of the fraction of active particles (see Information S2). We demonstrate this numerically in Information S2, thus showing that DA indeed works well provided it is implemented in the correct way. This implies that the main source of error in the past was the uncoupled approximation, and not DA itself.

It is thus of interest to develop and test numerical DA methods that efficiently and accurately approximate the dynamics of stochastic ion channels. Here we propose and test a DA implementation that gives the same results as MC modeling with two different models of neuronal excitability; to our knowledge the most thorough testing that any DA algorithm has been subjected to.

### Relation to Other Algorithms

Goldwyn *et al.* [33] tested the DA approach for coupled particles originally developed by Fox [29] and showed that a properly implemented DA can approach better the results of MC modeling, in the context of the HH model. However, they computed the square root of the diffusion matrix during execution, resulting in a slow computation speed. Another recent work by Linaro *et al.* [40] suggested an alternative DA implementation for the HH model, that uses similar equations to the uncoupled particles approach but with a noise term that is time-correlated in the way it should be when the particles are considered to be coupled. The correlation of the noise terms requires solving 7 (Na) or 4 (K) additional differential equations, of a complexity comparable to those presented here. Importantly, both works, as well as many others, employed a steady-state approximation for the calculation of the stochastic term matrix introduced. As we showed here, this approximation caused significant deviations in voltage clamp (Figure 1C) and the Rb model under current clamp (Figure 3), but not in the HH model under current clamp (Figure 6). Similarly, both previous methods (that use this approximation) also displayed deviations in voltage clamp and the current-clamped Rb (Figure 8), but not in the current-clamped HH (not shown). Note that both methods were previously tested in the HH setting ([33], [40]) – which may suggest why it was believed they were accurate. However, when the HH model was simulated using a fixed AP voltage trajectory (as in Figure 1 of [30]), we found that the steady-state approximation again introduces significant inaccuracies (see Information S3). Interestingly, this can be already spotted in [30], Figure 1– where the ‘V. clamp’ method (which employs the steady-state approximation) deviates from the exact results. Therefore, the steady-state approximation introduces inaccuracies in all the models we tested – albeit more strongly in the Rb model than in the HH model. It is important to note that among the channels that work on the time scale of action potentials, the sodium channel of the Rb model has fast kinetics (resembling channels from mammalian nodes of Ranvier), while the HH model possesses channels that are rather slow (giant squid axon at 6.3°C). Most likely, this is the reason why the Rb model is more affected by the steady-state approximation than the HH model. As the time scale relevant for models based in the mammalian nervous system is precisely that of the Rb model, our conclusions about the steady-state approximation are of importance for such models.

Both previous works [33], [40], as well as the original derivation by Fox [31], give specific instructions on how to construct the SDE for sodium and potassium channels, in the context of the HH model. However, generalizing these instructions to other kinetic schemes is not an easy task, even in the case where general expressions are given [40]. In contrast, our alternative derivation gave explicit, simple and general expressions for the both the diffusion matrix 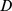 (eq. (10)) and its matrix square root 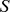 (eq. (13)). Interestingly, these results can be elegantly and succinctly described using the graph theory concepts – the graph here being the channel’s kinetic diagram, with each vertex corresponding to a channel state and each edge corresponding to a kinetic transition pair between two states. Then, 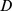 and 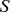 are straightforward generalizations of the Laplacian matrix and the incidence matrix [46], respectively, for the case of directed graphs with weighted edges (the weights being 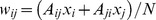). Note that the relation 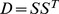 is then well known for unweighted directed graphs [46].

In order to compare with previous DA formulations [31], [33], we analytically found 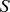 for the potassium case using Cholesky decomposition. Surprisingly, but in tune with our proposed equations, the resulting matrix was simpler (compare eq. (14) with (15)) and sparse (containing many zero elements). The exact and simple expression for 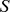 (eq. (13)) allowed us to avoid the use of the inaccurate steady state approximation and to improve simulation speed considerably. Specifically, instead of the 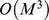 computational complexity of the numerical matrix square root implementation (as done in [33]) our method has a complexity between 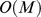 and 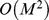, depending on the number of kinetic transitions (see eq. (13)). Numerically testing this, we observed our method run at least 20 times faster, depending on the software environment employed. Moreover, the equations that govern the dynamics of stochastic ion channels in our approach can be simply written as separate equations instead of matrix operations (e.g. eq. (16) for potassium and Information S1 for sodium). This facilitates their implementation in non matrix-oriented computation software such as Neuron, and may also simplify future analytical analysis of the behavior of the stochastic neuron.

We note a connection between the DA approach and another stochastic simulation method - the “binomial population” approach [47], [48], [49]. This approach employs eq. (11) directly, where each channel transition step 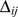 is distributed binomially. So essentially, the main additional approximation we made was that 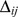 was a Gaussian RV. This can greatly reduce simulation speed since the generation of binomial RVs is much less efficient than Gaussian RVs, especially for large 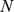 [50]. As noted, our simulations (as well as Goldwyn’s [33] and Linaro’s [40]) indicate that this approximation is very good, as long as 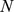 is not too small. However, if 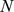 is small enough, so that the discrete nature of ion channel conductance becomes significant, then this approximation might break down. In that case, one can speculate that it might be more accurate to approximate 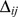 as a Poisson RV with parameter 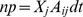 (by the law of rare events). Note that also in this approximation it is possible to pair opposing transition pairs 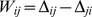 (as in eq. (12)) and generate 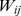 according to a Skellam distribution (the distribution of the difference between two Poisson RV). However, we have not investigated here whether or not the Poisson\Skellam approximations may actually improve the speed of binomial population algorithm or have any advantage over other methods (such as MC).

Finally, we note that a similar approach to ours was previously introduced in the field of chemical physics. As in our case, this equation, named “the *Chemical Langevin Equation*” (CLE) [51], [52], [53] sums the stochastic terms along transitions and not along states (compare our eq. (13) with eq. (23) in [51]). The main computational difference between that approach and ours is that we sum together the noise contributions from both directions of each transition pair (done in the conversion from eq. (11) to eq. (12)). This approximately halves the computation time, when the generation of pseudo-RVs is the main computational bottleneck. Conceptually, a comparison with CLE suggests that the DA approach might be extended to describe a more general setting than investigated here. For example, we could introduce a direct coupling between different channel types, via changes in ionic concentration-dependent channels (and not just voltage) or consider how the noisy kinetics of other complex cellular processes (such as ion channel regularization [30], [54]) can affect the neuronal response. However, in these cases, eq. (1) may not have a simple linear form.

### Numerical Efficiency

Following the practical approach of this work, we numerically evaluated the computational cost of three different algorithms: MC, our DA algorithm and the (inaccurate) uncoupled version of DA. In short, in almost all cases our DA approach significantly outperforms the MC approach. Also, our method only doubles the time required to solve the inaccurate, uncoupled version of DA (Figures 4 and 7). It also only doubles the time for solving the deterministic equations that ignore the stochastic terms (not shown).

Specifically, when comparing our DA to MC, in the Rb simulations (Figure 4C&D) the DA approach for coupled particles is at least an order of magnitude faster than MC for all values of 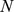 and 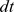 tested. In the HH simulations (Figure 7D&E) this remains true, except when low values of 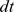 or 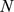 are used. Again we note that the results for Rb model are more significant to the mammalian nervous system, due to the similar kinetic timescales. Another issue to consider when comparing Figures 4 and 7 is that the Rb simulations presented here were performed in the Scilab numerical computation package while the HH simulations were implemented in NEURON. The latter will be always faster because it runs as compiled code; also variations in how each software implements numerical calculations at the processor level may cause further differences.

In all cases, however, the speed of simulations performed with the DA algorithm was only affected by the size of the integration time step and completely independent of the number of channels to be simulated, because the number of channels is only a parameter in the equations. On the contrary, MC modeling was heavily affected by the number of channels and less affected by the integration time step. In this case a greater number of channels imply more transitions per time step, and for each transition two new calculations have to be made, each requiring a new random number.

Thus, there will be situations where MC modeling may be numerically more efficient than DA. With a small number of channels there will be fewer transitions per time step and thus a MC simulation may run faster than a DA algorithm. This difference will be enhanced if the channels have slow kinetics, because this will reduce the probability of transitions. Also, if a small integration time step is required the DA algorithm can be as slow as MC modeling. In both these cases, it might be better to combine the MC and DA methods [55]: use MC for channel with slow kinetics, while handle the faster channels using the DA approach. The waterline between “slow” and “fast” timescales here would be the time step duration. Also, note that in the simulations presented here, random numbers were generated in simulation time. Further speed-up of the DA algorithm can be achieved by the use of a pre-generated random number list.

### Conclusions

This paper further confirms that the use of the Diffusion Approximation (DA), without any additional approximations, produce results that are in many ways indistinguishable from those of Markov Chain modeling (MC). Most importantly, we present the DA in a very simple, general and computationally efficient form, which will allow its easy implementation for any given kinetic scheme of a channel. We show that in the most common situations, the DA method proposed here has a numerical stability comparable to that of MC modeling (even with a simple Euler-Maruyama integration scheme), while being much faster. The fast simulation speed achieved makes conceivable its use in dynamic clamp experiments.

## Methods

### Models

To test the accuracy and efficiency of DA relative to MC modeling, both in their independent particles and coupled particles approaches, two models were employed in which different measures of simulation accuracy were calculated.

#### Mammalian node of ranvier – Rb model

The mammalian Node of Ranvier model [43] was the model employed previously to compare the performance of DA versus MC modeling [28], [41]. This model consists only of a voltage-dependent sodium channel and a voltage-independent leak current. The membrane current equation is.
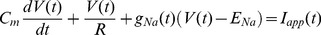
(18)with parameters *C_m_* = 18.9 nF; *R* = 7.372MΩ; 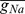 = 6.808 µS; *E_Na_* = 144 mV. The voltage is shifted so that the leak reversal potential is 0. The α and β transition rates are given by the following voltage dependent functions:



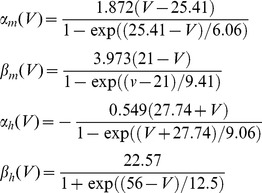
(19)Simulations of 1 ms were run in which a 100 µs current pulse was given at the beginning (Figure 2). The pulse amplitude varied between 5 and 6.5 pA. 1,000 simulations were run and the following parameters were calculated: *Firing efficiency*, the fraction of simulations in which an action potential was evoked; and the mean and the variance of *Firing time,* time at which the voltage reached or surpassed 80 mV. Firing efficiency versus pulse amplitude curve was fit to the cumulative Gaussian distribution.
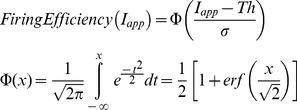



*erf(x)* represents the error function. *Th (threshold)* gives the amplitude for a probability of firing of 0.5, while σ quantifies the spread of the input/output relationship.

### Hodgkin and Huxley Model of Squid Giant Axon – HH Model

The original Hodgkin and Huxley [21] model was simulated with the equation.
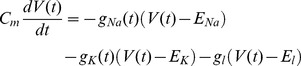
(20)


and parameters *C_m_* = 1 µF, *E_Na_* = 50 mV, *E_K_ = *-77 mV, *E_l_* = -54.4 mV, 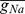 = 120 mS, 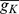 = 36 mS, *g_l_ = *0.3 mS (Voltages are shifted with respect to the original model to make the resting potential equal to -65 mV). The α and β functions employed are.
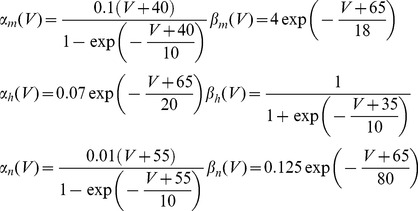
(21)


Simulations of 500 seconds were performed, and action potentials were recorded as the time at which the voltage reached or surpassed 0 mV. The time of action potentials during the simulation were stored, and the Inter-Spike Intervals (ISIs) were calculated. The normalized ISI distribution was fitted to an exponential decay function with a refractory period [16]:
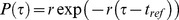
(22)


The first two values of the ISI distribution histogram were not included in the fitting procedure.

### Markov Chain Simulations of Coupled Gating Particles

There are two possible ways of implementing a coupled particles approach. The first consist of simulating 4 independent 2-state particles per channel, and a channel is considered open if and only if its four particles are in the open state. Therefore, the state of each particle (hence of each channel) must be tracked individually during the simulation [43].

In this paper a second approach is employed, that consists in building a multi-state MC per channel considering the possible combinations of active particles. This allows for the faster number-tracking algorithm employed for simulations [16], [26], [27]. Given that particles of a given kind are identical and independent, a Sodium channel has 8 possible states while a Potassium channel has 5 states:



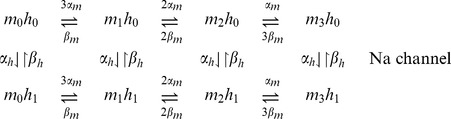


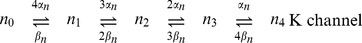



In this approach, only one state of each MC represents the conducting or open channel, which is the state with all particles active (*m*_3_ *h*_1_ or *n*_4_). Then the conductance is calculated with the fraction of channels or MCs that are in the open state:
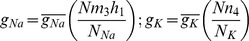
where *Nm_3_h_1_* and *Nn_4_* are the number of channels in the state *m_3_h_1_* and *n_4_*, respectively.

#### Diffusion approximation

The DA for channels with coupled gating particles is detailed in the Results section.

### Numerical Implementations

#### Software implementation

All models and algorithms were implemented in Scilab, a matrix-oriented numerical software (www.scilab.org), and NEURON, a simulation environment oriented to the modeling neurons and neural networks (www.neuron.yale.edu). Source files and scripts are available in ModelDB (http://senselab.med.yale.edu/ModelDB/). Both environments produced identical results but simulations in NEURON run faster because it runs in compiled mode. Results presented here (most importantly, processing time data) correspond to simulations in Scilab for the mammalian Node of Ranvier (Rb) model and simulations in NEURON for the squid giant axon (HH) model.

#### Markov chain modeling

Independent MCs were modeled using a number-tracking algorithm [16], [26], [27], [28]. Thoroughly described in [28], briefly this algorithm consist in keeping track of the number of MCs in each state, rather than keeping track of each MC individually. At any time t, the probability density function of the lifetime before the next transition (any transition) is.
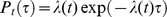
where λ(*t*) is the effective transition rate given by



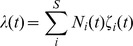
where *S* is the total number of states in the MC, *N_i_* is the number of MCs in state *i*, and ζ(*t*) is the sum of transition rates escaping from state *i*. If there is more than one type of MC, they are all summed into λ. The time of the next transition *t_n_* is calculated by drawing a random number uniformly distributed within [0,1] and taking the inverse of the c.d.f. of the lifetime. If *t_n_*≤*t*, a transition has to be calculated before updating the current equation. Among all possible transitions, the probability of transition *j* to occur is



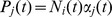
where *i* is the state originating the transition *j* and α*_j_* its rate. A cumulative probability for all transitions is calculated and a transition is chosen by drawing a random number uniformly distributed within [0,1]. The number of MCs at each state is updated, and a new time for the next transition is calculated. When no more transitions are to occur in the current time step, the current equation is advanced one time step using an Euler integration scheme.

#### Diffusion approximation

Stochastic differential equations for DA were solved by an Euler-Maruyama integration method. For the coupled particles approach, a better numerical stability is obtained if the fact that the sum of state variables for a given channel is 1 is taken into account, also reducing the number of SDEs to be solved. Thus, for potassium channels the equations used for advancing one time step are
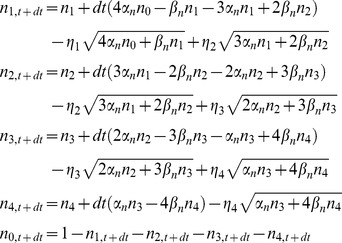



being η_1_, η_2_, η_3_, and η_4_ independent Gaussian RVs with zero mean and standard deviation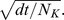 *n*_0_– *n*_4_ stand for *n*_0,t_ – *n*_4,t_, i.e. the value of the variables at time *t*. A similar set of equations was used for sodium channels.

No rounding was performed on the variables, nor were they bound to lie between 0 and 1 (see *Numerical implementation issues* section in Results). To ensure real valued random terms, the square roots were applied to the absolute value of the operand.

For the steady state approximation, the variables *n_i_* and *m_i_h_j_* were replaced by their steady state values in all the noise terms:
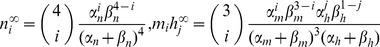
(23)


## Supporting Information

Information S1 **An intuitive set of rules for constructing the SDEs corresponding to any given kinetic scheme.** Also the full set of SDEs employed for the 8-state Sodium channel is presented.
(PDF)

Information S2 **Uncoupled Markov Chain simulations yield the same numerical results as the uncoupled Diffusion Approximation.**
(PDF)

Information S3 **A second Voltage-clamp comparison of the simulation algorithms, in which the HH stochastic channels are subject to a fixed voltage trajectory containing an action potential.**
(PDF)
