# Supplementary material for: Simple, Fast and Accurate Implementation of the Diffusion Approximation Algorithm for Stochastic Ion Channels with Multiple States
Source: PLoS One. 2012 May 22;7(5):e36670. doi: 10.1371/journal.pone.0036670 (PMC3358312; doi:10.1371/journal.pone.0036670)
Supplement: Information S1 — An intuitive set of rules for constructing the SDEs corresponding to any given kinetic scheme. Also the full set of SDEs employed for the 8-state Sodium channel is presented. (PDF) [file pone.0036670.s001.pdf]

SUPPORTING INFORMATION S1

Method for building the SDE system

Here we explain intuitively the method for building the stochastic differential equations (SDE) that will approximate any kinetic scheme for an ion channel, and derive the SDE for a sodium channel. We take as a working example the m_1h_0 state from the eight-state kinetic scheme for sodium channels:

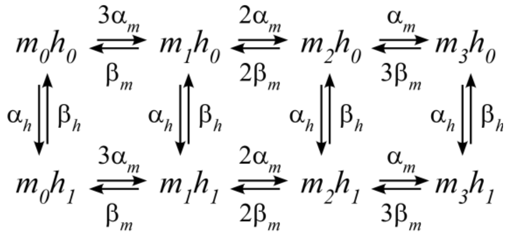

Deterministic terms

For the deterministic (drift) terms of the stochastic differential equation, the six transitions that go from or come to the m_1h_0 state have to be considered. Each arrow represents a possible transition, its probability given by the product of the voltage-dependent kinetic constant times the value of the state that is at the beginning of the arrow. Terms given by arrows starting at m_1h_0 are negative:

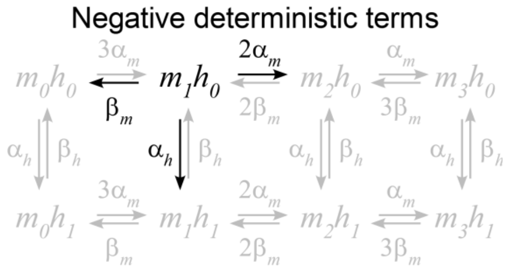

while the terms given by the arrows that end at m_1h_0 are positive:

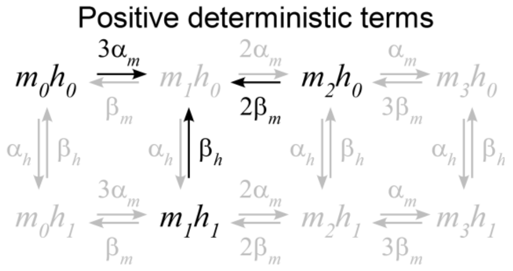

Thus, the six deterministic terms related to m_1h_0 are:

$$3\alpha_m m_0 h_0 - \beta_m m_1 h_0 - 2\alpha_m m_1 h_0 + 2\beta_m m_2 h_0 - \alpha_h m_1 h_0 + \beta_h m_1 h_1$$
$$= 3\alpha_m m_0 h_0 + 2\beta_m m_2 h_0 + \beta_h m_1 h_1 - \left(\beta_m + 2\alpha_m + \alpha_h\right) m_1 h$$

Stochastic terms

For the stochastic terms the transition arrows are to be considered in pairs:

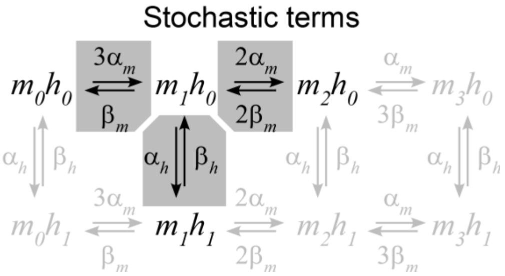

The pairs that are connected to m_1h_0 are:

$$3\alpha_m m_0 h_0 \; / \; \beta_m m_1 h_0$$
$$2\alpha_m m_1 h_0 \; / \; 2\beta_m m_2 h_0$$
$$\alpha_h m_1 h_0 \; / \; \beta_h m_1 h_1$$

Each pair of arrows originates a random term with zero mean and standard deviation equal to the square root of the sum of the transition probabilities, divided by the square root of N_{Na}, the number of sodium channels. In this case, all terms are considered positive. For the m_1h_0 state, the stochastic terms are

$$\xi_1\sqrt{\frac{3\alpha_m m_0 h_0 + \beta_m m_1 h_0}{N_{Na}}} + \xi_2\sqrt{\frac{2\alpha_m m_1 h_0 + 2\beta_m m_2 h_0}{N_{Na}}} + \xi_3\sqrt{\frac{\alpha_h m_1 h_0 + \beta_h m_1 h_1}{N_{Na}}}$$

where ξ_1, ξ_2 and ξ_3 are independent Gaussian white noise terms with zero mean and unit variance. Note that each pair of arrows connects two (and only two) states. In the SDE for the second of such states, the stochastic term has to be repeated exactly (the same Gaussian term) but with the opposite sign. For instance, the transition pair $2\alpha_m m_1 h_0/2\beta_m m_2 h_0$ connects m_1h_0 and m_2h_0; therefore the SDE for the m_2h_0 state must contain the term

$$-\xi_2\sqrt{\frac{2\alpha_m m_1 h_0 + 2\beta_m m_2 h_0}{N_{Na}}},$$

repeating the same Gaussian white noise term as in the m_1h_0 equation but with opposite sign. (being Gaussian terms with zero mean it doesn't matter which one goes positive; the key point is to have the term positive in one equation and negative in the other). Because of this, the full set of equations for the sodium channels has 20 stochastic terms but only 10 random variables; analogously the equations for a 5-state potassium channels have 8 stochastic terms with 4 random variables.

Following this procedure while keeping care of repeating stochastic term with opposite signs, the following set of equations for the sodium channel is obtained:

$$
\begin{aligned}
\frac{dm_0h_0}{dt} &= \left(-3\alpha_m m_0 h_0 + \beta_m m_1 h_0 - \alpha_h m_0 h_0 + \beta_h m_0 h_1\right) \\
&\quad + \xi_1 \frac{1}{\sqrt{N_{Na}}} \sqrt{3\alpha_m m_0 h_0 + \beta_m m_1 h_0} + \xi_4 \frac{1}{\sqrt{N_{Na}}} \sqrt{\alpha_h m_0 h_0 + \beta_h m_0 h_1}
\end{aligned}
$$

$$
\begin{aligned}
\frac{dm_1h_0}{dt} &= \left(3\alpha_m m_0 h_0 - \beta_m m_1 h_0 - 2\alpha_m m_1 h_0 + 2\beta_m m_2 h_0 - \alpha_h m_1 h_0 + \beta_h m_1 h_1\right) \\
&\quad - \xi_1 \frac{1}{\sqrt{N_{Na}}} \sqrt{3\alpha_m m_0 h_0 + \beta_m m_1 h_0} + \xi_2 \frac{1}{\sqrt{N_{Na}}} \sqrt{2\alpha_m m_1 h_0 + 2\beta_m m_2 h_0} + \xi_5 \frac{1}{\sqrt{N_{Na}}} \sqrt{\alpha_h m_1 h_0 + \beta_h m_1 h_1}
\end{aligned}
$$

$$
\begin{aligned}
\frac{dm_2h_0}{dt} &= \left(2\alpha_m m_1 h_0 - 2\beta_m m_2 h_0 - \alpha_m m_2 h_0 + 3\beta_m m_3 h_0 - \alpha_h m_2 h_0 + \beta_h m_2 h_1\right) \\
&\quad - \xi_2 \frac{1}{\sqrt{N_{Na}}} \sqrt{2\alpha_m m_1 h_0 + 2\beta_m m_2 h_0} + \xi_3 \frac{1}{\sqrt{N_{Na}}} \sqrt{\alpha_m m_2 h_0 + 3\beta_m m_3 h_0} + \xi_6 \frac{1}{\sqrt{N_{Na}}} \sqrt{\alpha_h m_2 h_0 + \beta_h m_2 h_1}
\end{aligned}
$$

$$
\begin{aligned}
\frac{dm_3h_0}{dt} &= \left(\alpha_m m_2 h_0 - 3\beta_m m_3 h_0 - \alpha_h m_3 h_0 + \beta_h m_3 h_1\right) \\
&\quad - \xi_3 \frac{1}{\sqrt{N_{Na}}} \sqrt{\alpha_m m_2 h_0 + 3\beta_m m_3 h_0} + \xi_7 \frac{1}{\sqrt{N_{Na}}} \sqrt{\alpha_h m_3 h_0 + \beta_h m_3 h_1}
\end{aligned}
$$

$$
\begin{aligned}
\frac{dm_0h_1}{dt} &= \left(-3\alpha_m m_0 h_1 + \beta_m m_1 h_1 + \alpha_h m_0 h_0 - \beta_h m_0 h_1\right) \\
&\quad + \xi_8 \frac{1}{\sqrt{N_{Na}}} \sqrt{3\alpha_m m_0 h_1 + \beta_m m_1 h_1} - \xi_4 \frac{1}{\sqrt{N_{Na}}} \sqrt{\alpha_h m_0 h_0 + \beta_h m_0 h_1}
\end{aligned}
$$

$$
\begin{aligned}
\frac{dm_1h_1}{dt} &= \left(3\alpha_m m_0 h_1 - \beta_m m_1 h_1 - 2\alpha_m m_1 h_1 + 2\beta_m m_2 h_1 + \alpha_h m_1 h_0 - \beta_h m_1 h_1\right) \\
&\quad - \xi_8 \frac{1}{\sqrt{N_{Na}}} \sqrt{3\alpha_m m_0 h_1 + \beta_m m_1 h_1} + \xi_9 \frac{1}{\sqrt{N_{Na}}} \sqrt{2\alpha_m m_1 h_1 + 2\beta_m m_2 h_1} - \xi_5 \frac{1}{\sqrt{N_{Na}}} \sqrt{\alpha_h m_1 h_0 + \beta_h m_1 h_1}
\end{aligned}
$$

$$
\begin{aligned}
\frac{dm_2h_1}{dt} &= \left(2\alpha_m m_1 h_1 - 2\beta_m m_2 h_1 - \alpha_m m_2 h_1 + 3\beta_m m_3 h_1 + \alpha_h m_2 h_0 - \beta_h m_2 h_1\right) \\
&\quad - \xi_9 \frac{1}{\sqrt{N_{Na}}} \sqrt{2\alpha_m m_1 h_1 + 2\beta_m m_2 h_1} + \xi_{10} \frac{1}{\sqrt{N_{Na}}} \sqrt{\alpha_m m_2 h_1 + 3\beta_m m_3 h_1} - \xi_6 \frac{1}{\sqrt{N_{Na}}} \sqrt{\alpha_h m_2 h_0 + \beta_h m_2 h_1}
\end{aligned}
$$

$$
\begin{aligned}
\frac{dm_3h_1}{dt} &= \left(\alpha_m m_2 h_1 - 3\beta_m m_3 h_1 + \alpha_h m_3 h_0 - \beta_h m_3 h_1\right) \\
&\quad - \xi_{10} \frac{1}{\sqrt{N_{Na}}} \sqrt{\alpha_m m_2 h_1 + 3\beta_m m_3 h_1} - \xi_7 \frac{1}{\sqrt{N_{Na}}} \sqrt{\alpha_h m_3 h_0 + \beta_h m_3 h_1}
\end{aligned}
$$
