# Supplementary material for: Simple, Fast and Accurate Implementation of the Diffusion Approximation Algorithm for Stochastic Ion Channels with Multiple States
Source: PLoS One. 2012 May 22;7(5):e36670. doi: 10.1371/journal.pone.0036670 (PMC3358312; doi:10.1371/journal.pone.0036670)
Supplement: Information S2 — Uncoupled Markov Chain simulations yield the same numerical results as the uncoupled Diffusion Approximation. (PDF) [file pone.0036670.s002.pdf]

SUPPORTING INFORMATION S2

The uncoupled DA and its Markov Chain equivalent

The inaccuracy of the uncoupled form of the Diffusion Approximation algorithm to represent stochastic channels with multiple gating particles has been extensively studied numerically and analytically [1,2,3,4,5]. As shown analytically by [5], the uncoupled DA is accurately representing *another* type of MC modeling. To confirm this numerically that this is the case (i.e. the DA approximation works also in the uncoupled scenario) we compared the uncoupled particles DA algorithm with an uncoupled version of the MC modeling. This was to show that the uncoupled DA implementation behaves as independent, two-state MCs where the conductance is calculated over the fraction of active gating particles (see Figure S1).

Figure S2A shows results of the Rubinstein's Node of Ranvier model testing for both coupled and uncoupled MCs and DA algorithms. See Figure 2B and 2D in the main text for a description of left and middle panels, respectively; and Figure 3 for the right panel. Figure S2B shows results of the spontaneous action potential firing observed in the HH model with the uncoupled and coupled versions of DA and MC modeling. Figure description is as in Figures 6A and 6D of the main text. These results confirm that the uncoupled DA perfectly matches the behavior of uncoupled MCs in both models under current clamp testing. As we explain in Supporting Information S3, this is also true for voltage clamp simulations (Figure S3D and S3F). Thus, given that the DA is appropriately implemented, it will always approximate very closely the behavior of exact Markov Chain modeling; what makes a difference is the coupling or uncoupling of gating particles.

We want to note that to simulate uncoupled MCs not only means to switch from N 5-state (K) or 8-state (Na) to 4N 2-state MCs. If the conductance is calculated as the fraction of *channels* that have all gating particles active, then this is a coupled particles scenario. However, this type of simulation requires keeping track of each gating particle and channeling individually [6], making impossible to apply the efficient channel number tracking algorithm employed here (see Methods and [7]). Our definition of uncoupled MCs implies that the conductance is calculated over the fraction of active *particles* (Figure S1), an approach that may seem wrong when in the context of MCs but that nevertheless is exactly what the uncoupled DA is representing.

Methods: Simulation of Uncoupled independent particles

N channels are simulated as $4N$ independent, 2-state particles:

$$a_0 \underset{\beta_a}{\overset{\alpha_a}{\rightleftarrows}} a_1 \qquad (a = n,m,h)$$

where α_a is the transition probability from the 0 to the 1 state, and β_a the transition probability from the 1 to the 0 state. N_{Na} Sodium channels are simulated as $3N_{Na}$ m particles and N_{Na} h particles, and at each time step the sodium conductance is calculated as

$$g_{Na} = \overline{g_{Na}} \left(\frac{Nm_1}{3N_{Na}} \right)^3 \left(\frac{Nh_1}{N_{Na}} \right). \tag{1}$$

where Nm_1 and Nh_1 are the number of m and h particles in the '1' (active) state, respectively. N_K Potassium channels are simulated as $4N_K$ n particles and the potassium conductance is calculated as

$$g_K = \overline{g_K}\left(\frac{Nn_1}{4N_K}\right)^4 . \tag{2}$$

Nn_1 is the number of n particles that are in the '1' state.

Diffusion Approximation

The DA in the case of independent particles uses the variables $m, h, n \in [0,1]$ to keep track of the fraction of m, h, and n particles, respectively, that are in the '1' state. It follows immediately that the fraction of particles in the '0' state will be 1-m, 1-h, and 1-n, respectively. Fox and Lu [8] showed that the time evolution of the variables is given by the SDE

$$\frac{da}{dt} = \alpha_a(1-a) - \beta_a a + \sigma_a(t)\xi(t) \tag{3}$$

where a represents either m, h or n. The stochastic term ξ(t) is a Gaussian white noise with zero mean and unit variance that is scaled by σ_a(t), being

$$\sigma_a(t) = \sqrt{\frac{\alpha_a(1-a)+\beta_a a}{N_a}} \tag{4}$$

where N_a is the number of a particles (N_m=3N_{Na}, N_h=N_{Na} and N_a=4N_K). When the steady state approximation was used, the noise scaling factor was calculated as

$$\sigma_a(t) = \sqrt{\frac{2\alpha_a\beta_a}{N_a(\alpha_a+\beta_a)}} \tag{5}$$

The conductance of sodium and potassium are calculated using the classical Hodgkin & Huxley expressions

$$g_{Na} = \overline{g_{Na}}m^3h \quad \text{and} \quad g_K = \overline{g_K}n^4 .$$

References

1. Linaro D, Storace M, Giugliano M (2011) Accurate and fast simulation of channel noise in conductance-based model neurons by diffusion approximation. PLoS Comput Biol 7: e1001102.
2. Bruce IC (2007) Implementation issues in approximate methods for stochastic Hodgkin-Huxley models. Ann Biomed Eng 35: 315-318; author reply 319.
3. Bruce IC (2009) Evaluation of stochastic differential equation approximation of ion channel gating models. Ann Biomed Eng 37: 824-838.
4. Goldwyn JH, Shea-Brown E (2011) The what and where of adding channel noise to the hodgkin-huxley equations. PLoS Comput Biol 7: e1002247.
5. Goldwyn JH, Imennov NS, Famulare M, Shea-Brown E (2011) Stochastic differential equation models for ion channel noise in Hodgkin-Huxley neurons. Phys Rev E Stat Nonlin Soft Matter Phys 83: 041908.
6. Rubinstein JT (1995) Threshold fluctuations in an N sodium channel model of the node of Ranvier. Biophys J 68: 779-785.
7. Mino H, Rubinstein JT, White JA (2002) Comparison of algorithms for the simulation of action potentials with stochastic sodium channels. Ann Biomed Eng 30: 578-587.
8. Fox RF, Lu Y (1994) Emergent collective behavior in large numbers of globally coupled independently stochastic ion channels. Phys Rev E Stat Phys Plasmas Fluids Relat Interdiscip Topics 49: 3421-3431.

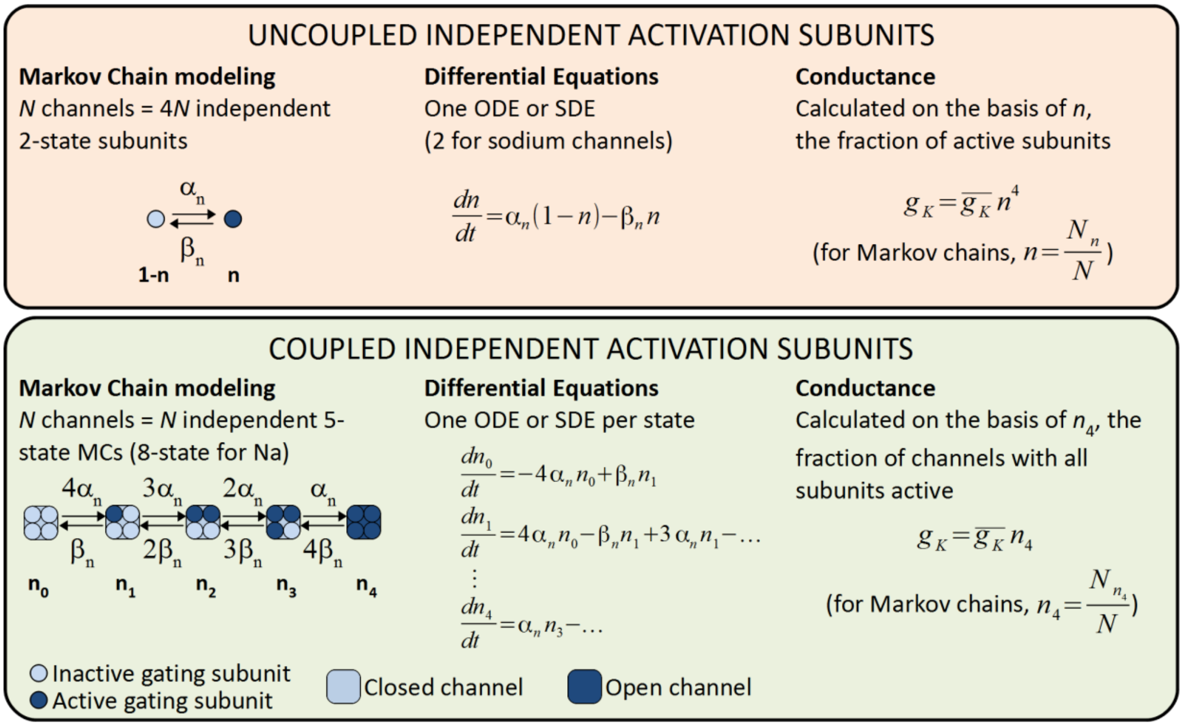
UNCOUPLED INDEPENDENT ACTIVATION SUBUNITS
Markov Chain modeling
N channels = 4N independent 2-state subunits
α_n
β_n
1-n
n
Differential Equations
One ODE or SDE (2 for sodium channels)
$\frac{dn}{dt} = \alpha_n(1-n) - \beta_n n$
Conductance
Calculated on the basis of n, the fraction of active subunits
$g_K = \overline{g_K} n^4$
(for Markov chains, $n = \frac{N_n}{N}$)
COUPLED INDEPENDENT ACTIVATION SUBUNITS
Markov Chain modeling
N channels = N independent 5-state MCs (8-state for Na)
$4\alpha_n$
$3\alpha_n$
$2\alpha_n$
α_n
β_n
$2\beta_n$
$3\beta_n$
$4\beta_n$
n_0
n_1
n_2
n_3
n_4
Differential Equations
One ODE or SDE per state
$\frac{dn_0}{dt} = -4\alpha_n n_0 + \beta_n n_1$
$\frac{dn_1}{dt} = 4\alpha_n n_0 - \beta_n n_1 + 3\alpha_n n_1 - \dots$
$\vdots$
$\frac{dn_4}{dt} = \alpha_n n_3 - \dots$
Conductance
Calculated on the basis of n_4, the fraction of channels with all subunits active
$g_K = \overline{g_K} n_4$
(for Markov chains, $n_4 = \frac{N_{n_4}}{N}$)
Inactive gating subunit
Active gating subunit
Closed channel
Open channel

Figure S1

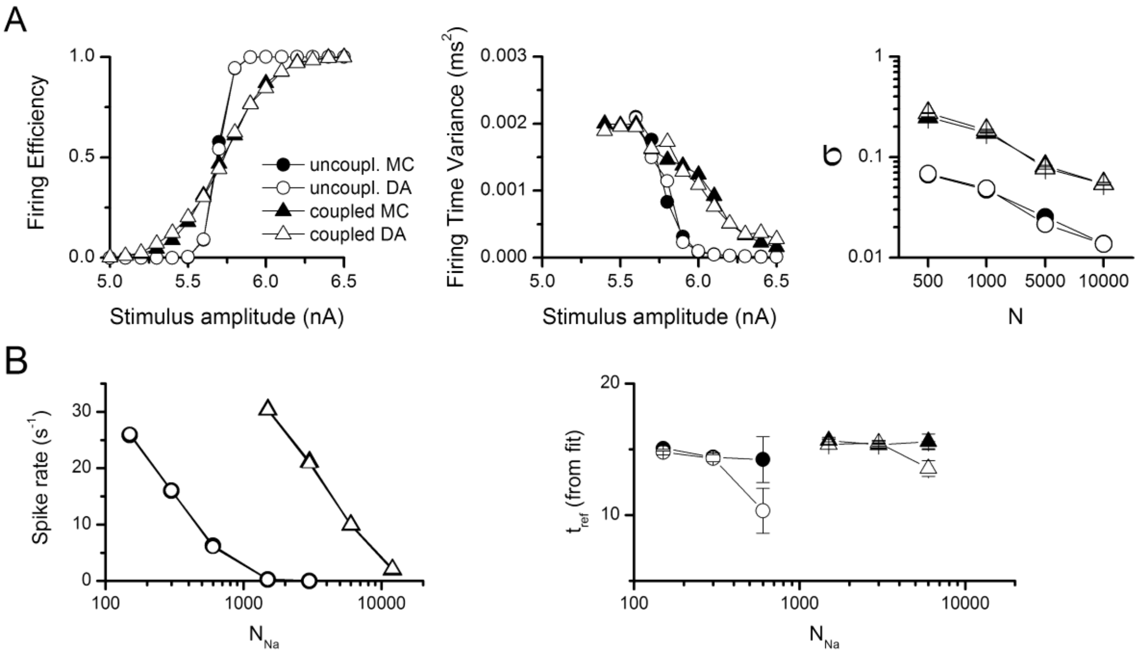

A
Firing Efficiency
Stimulus amplitude (nA)
uncoupl. MC
uncoupl. DA
coupled MC
coupled DA
Firing Time Variance (ms²)
σ
N
B
Spike rate (s⁻¹)
N_Na
t_ref (from fit)

Figure S2
