# Supplementary material for: Simple, Fast and Accurate Implementation of the Diffusion Approximation Algorithm for Stochastic Ion Channels with Multiple States
Source: PLoS One. 2012 May 22;7(5):e36670. doi: 10.1371/journal.pone.0036670 (PMC3358312; doi:10.1371/journal.pone.0036670)
Supplement: Information S3 — A second Voltage-clamp comparison of the simulation algorithms, in which the HH stochastic channels are subject to a fixed voltage trajectory containing an action potential. (PDF) [file pone.0036670.s003.pdf]

SUPPORTING INFORMATION S3

Simulations with a fixed voltage trajectory

We tested the response of the different HH models to a fixed voltage trajectory, obtained from a spontaneous action potential of the coupled MC model (coupled gating particles, Markov Chain modeling) with 6000 Na channels and 1800 K channels. Figure S3A shows the voltage trajectory. It is characterized by a period of subthreshold voltage fluctuations that eventually lead to the firing of an action potential. This voltage trace was used as input for the different stochastic models in 2,000 independent voltage-clamp runs (5000 for MC models), and the resulting fraction of open channels (Na and K) was recorded. Then, the mean and variance for each time point was calculated. Also, the same simulation was performed with the deterministic HH model, and the mean and variance were calculated as [1]:

$$E\left[Na_O\right] = m^3h \qquad\qquad E\left[K_O\right] = n^4$$

$$\mathrm{var}\left[Na_O\right] = \frac{m^3h\left(1-m^3h\right)}{N_{Na}} \qquad\qquad \mathrm{var}\left[K_O\right] = \frac{n^4\left(1-n^4\right)}{N_K}.$$

These values were used as the 'exact solution' to which the results of the simulations were compared. Figure S3B shows the time course of mean fraction of open channels that was essentially the same for all simulations (only minor differences were observed in the third or fourth significant digit).

Figure S3C shows the time course of the variance of open Na channels for the coupled particles models along with the exact solution. While it is very hard to spot any difference between the exact, coupled Markov Chains (MC) and coupled diffusion approximation (DA) models, the diffusion approximation with steady-state variable values for the random terms (DAss) clearly underestimates the variance of open sodium channels, a deviation that is higher during the action potential (right inset). Regarding the models with uncoupled gating particles, Figure S3D shows that both the Markov Chains model (uncoupl. MC) and the diffusion approximation (uncoupl. DA) heavily underestimate the variance during the subthreshold regime. During the action potential, the deviation is larger for the DA with steady-state approximation (uncoupl. DAss).

Very similar observations can be made with the time course of the variance of open potassium channels. Figure S3E shows that Markov Chains and diffusion approximation with coupled particles are indistinguishable from the exact solution. Once again, when the steady-state approximation is used for the random terms of DA, an important deviation is observed. However, in this case the variance is sometimes underestimated and sometimes overestimated. Finally, Figure S3F shows that uncoupling of gating particles results in underestimation of the variance for all the models with respect to the exact solution, again being the steady state approximation the model that deviates the most. Note, however, that the uncoupled MC and DA models are almost indistinguishable.

In summary, this voltage clamp test produced the same findings as in Figures 6 and 7 from the paper, namely that the DA implementation that we present reproduces quite accurately the

behavior of Markov Chains representing coupled gating particles. On the other hand, uncoupled DA reproduces uncoupled Markov Chains behavior (as in Supporting Information S2). Also, the steady-state approximation introduces severe deviations in both the coupled and uncoupled algorithms.

Two observations have to be made regarding the results presented by Goldwyn and Shea-Brown [1] using this voltage clamp simulation (Figure 1 there). First, the coupled particles DA algorithm that they implemented (Syst. Size in Figure 1 there) does *not* make use of the steady state approximation unlike the previous work by Goldwyn *et al.* [2] which does. Although not mentioned in [1], this can be verified by comparing the codes available at ModelDB (http://www.senselab.yale.edu/modeldb, accessions 128502 and 138950, respectively). Because of that, the coupled particles DA presented in [2] corresponds with our coupled DAss model, while the DA presented in [1] corresponds with our coupled DA model and as expected follows quite well the exact solution. However note that the DA as implemented by Goldwyn and Shea-Brown [1] is extremely slow when compared with ours and demands matrix square root computations.

Second, the variance of open K channels for the *uncoupled* subunits model in [1] ('Subunit' model in Figure 1 there) is about four times the variance of our uncoupled model (HH2MC or HH2DA). This is because when Goldwyn and Shea-Brown implement this DA for the potassium channel activation particle (*n*) as

$$dn = \left(\alpha_n\left(1-n\right)-\beta_n n\right)dt+\sqrt{\frac{\alpha_n\left(1-n\right)+\beta_n n}{N_K}}dW_t\,,$$

while our implementation (see eq. 4 above) uses the number of *n* particles ($4N_K$) in the variance term. Thus, it is expected that we obtain a variance that is approximately ¼ of that obtained by them. We think our implementation is the correct because what this algorithm approximates is the behavior of a number of independent *gating particles*, and not channels (in fact it is also called subunit or particle approach) and this number is four times the number of channels. The comparison with the HH2MC model (as well as Rb2MC vs. Rb2DA) makes evident that this is the case. Nevertheless, the inadequacy of either algorithm (dividing by N_K or $4N_K$) to model channels with multiple gating particles makes this issue irrelevant.

References

1. Goldwyn JH, Shea-Brown E (2011) The what and where of adding channel noise to the hodgkin-huxley equations. PLoS Comput Biol 7: e1002247.
2. Goldwyn JH, Imennov NS, Famulare M, Shea-Brown E (2011) Stochastic differential equation models for ion channel noise in Hodgkin-Huxley neurons. Phys Rev E Stat Nonlin Soft Matter Phys 83: 041908.

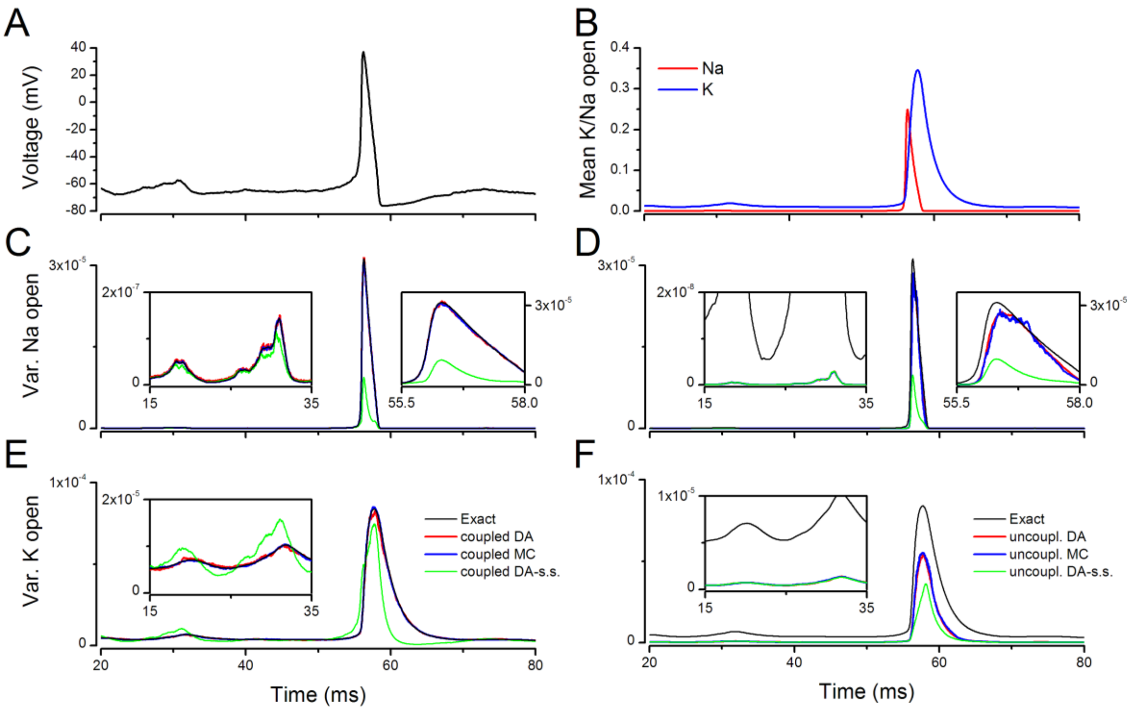
A
B
C
D
E
F
Voltage (mV)
Mean K/Na open
Na
K
Var. Na open
Var. K open
Exact
coupled DA
coupled MC
coupled DA-s.s.
uncoupl. DA
uncoupl. MC
uncoupl. DA-s.s.
Time (ms)

Figure S3
